# Intra-Platform Repeatability and Inter-Platform Comparability of MicroRNA Microarray Technology

**DOI:** 10.1371/journal.pone.0005540

**Published:** 2009-05-14

**Authors:** Fumiaki Sato, Soken Tsuchiya, Kazuya Terasawa, Gozoh Tsujimoto

**Affiliations:** 1 Department of Nanobio Drug Discovery, Graduate School of Pharmaceutical Sciences, Kyoto University, Kyoto, Kyoto, Japan; 2 Department of Pharcogenomics, Graduate School of Pharmaceutical Sciences, Kyoto University, Kyoto, Kyoto, Japan; Keio University, Japan

## Abstract

Over the last decade, DNA microarray technology has provided a great contribution to the life sciences. The MicroArray Quality Control (MAQC) project demonstrated the way to analyze the expression microarray. Recently, microarray technology has been utilized to analyze a comprehensive microRNA expression profiling. Currently, several platforms of microRNA microarray chips are commercially available. Thus, we compared repeatability and comparability of five different microRNA microarray platforms (Agilent, Ambion, Exiqon, Invitrogen and Toray) using 309 microRNAs probes, and the Taqman microRNA system using 142 microRNA probes. This study demonstrated that microRNA microarray has high intra-platform repeatability and comparability to quantitative RT-PCR of microRNA. Among the five platforms, Agilent and Toray array showed relatively better performances than the others. However, the current lineup of commercially available microRNA microarray systems fails to show good inter-platform concordance, probably because of lack of an adequate normalization method and severe divergence in stringency of detection call criteria between different platforms. This study provided the basic information about the performance and the problems specific to the current microRNA microarray systems.

## Introduction

Since the first DNA microarray paper demonstrated that microarray technology can monitor multiple gene expression profile in 1995 [1], DNA microarray technology has been developed steadily. After the Human Genome Project was finished, the ability of DNA microarray expanded to genome-wide analysis of not only gene expression profiling, but also, genome variation, epigenetics, DNA-protein interaction, and so on. In the research field, these genome-wide analyses using microarray technology have been providing deeper biological insights for a decade. In the clinical field, the US Food and Drug Administration (FDA) approved MammaPrint® as the first *in vitro* diagnostic multivariate index assay (IVDMIA) in February, 2007. Thus, microarray-based transcriptome devices started to be utilized to stratify patients for personalized medicine. For the quality control and standardization of microarray chips, the US FDA initiated the MicroArray Quality Control project (MAQC) in 2005. A series of reports regarding the first phase of the MAQC project was published in 2006 [2]–[7]. The MAQC report showed intra platform consistency across test sites as well as a high level of inter-platform concordance in terms of genes identified as differentially expressed.

MicroRNAs are a class of small non-coding RNAs [19–23 nucleotides (nt)] that have been found in animal and plant cells. As of today, 718 human microRNAs are registered in the miRBase database (Release 13, March, 2009) [8]–[11]. MicroRNA genes are transcribed as non-coding transcripts, and processed through a series of sequential steps involving the RNase III enzymes, Drosha and Dicer. The processed microRNAs are finally incorporated into the RNA-induced silencing complex (RISC) to mediate target mRNA repression of translation and/or degradation. It is reported that microRNAs are involved in physiological and pathological functions, such as the regulation of developmental timing and pattern formation [12], restriction of differentiation potential [13], chromatin rearrangements [14], and carcinogenesis [15]. Many of the mechanistic details still remain unknown.

Recently, microarray technology has been utilized to analyze a comprehensive microRNA expression profiling. Currently, several platforms of microRNA microarray chips are commercially available. As mentioned above, the MAQC Project is currently underway for quality control and standardization of mRNA expression microarray. However, no comparative and quality control study of microRNA microarray platforms has been reported yet. Therefore, we compared repeatability and comparability of microRNA microarray using five different platforms (Agilent, Ambion, Exiqon, Invitrogen and Toray). In addition, we compared quantitivity of microarray data generated from five different platforms with that of quantitative RT-PCR (Taqman) method, which is the golden standard method of microRNA measurement.

## Results

### Experimental design

**Figure 1:**
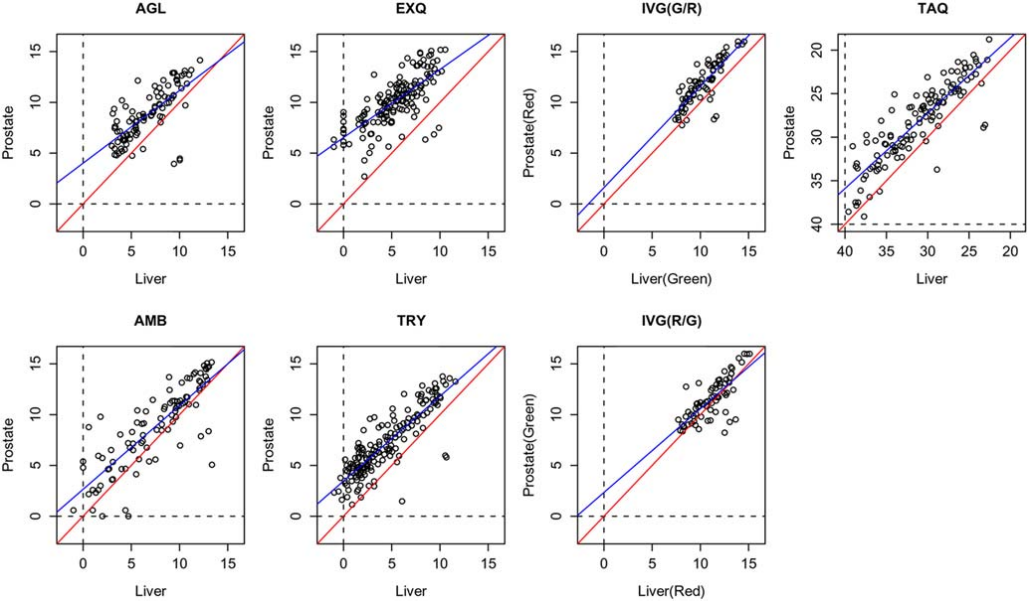
MicroRNA expression level in human liver and prostate tissues. For the microarray platforms, log2 transformed values of representative signal intensity for detection call-positive microRNAs were plotted. For the Taqman analysis, Ct values of microRNAs were plotted. Red and blue lines indicate Y = X line and regressed linear line, respectively. In all scatter plots. blue lines were shifted upward, which indicated that the general microRNA expression level in human prostate was higher than in human liver.

This project repeatedly assayed two RNA sample types on a variety of microRNA expression platforms at one laboratory. Our preliminary experiments showed that the amount of microRNA obtained from the same amount of total RNA depends on the tissue types of the samples (data not shown). This finding suggested that repeatability or comparability of microRNA microarray analysis might depend on the amount of microRNA contained in total RNA. To assess the reproducibility of microRNA microarray data using the different tissue types, we chose both tissue samples, which contain relatively small and large amounts of microRNA. Our preliminary data shows that mouse liver tissue contains relatively small amounts of microRNAs. Therefore, we used two types of total RNA, FirstChoice® Human Liver Total RNA (Ambion, lot no. 040000129) and FirstChoice® Human Prostate Total RNA (Ambion, lot no. 050500710), in this study. In fact, the amount of microRNAs in Human Liver Total RNA was smaller than that of Human Prostate Total RNA (Figure 1).

Five commercially available microRNA microarray platforms were tested: Agilent Technologies (AGL); Ambion Inc. (AMB); Exiqon (EXQ); Invitrogen (IVG) and Toray Industries Inc. (TRY) (Table 1). Four of the microarray providers used one-color protocols where one labeled RNA sample was hybridized to each microarray. The Invitrogen array was tested using a two-color and dye-swapping protocol so that, at first, two RNA samples were divided and differently labeled in red-green and green-red combinations, and each combination of the RNA sample set was simultaneously hybridized to a microarray.

**Table 1:** microRNA expression platforms and experimental procedures.

Code	Protocol	Platform	miRBase release	# of miRNAs	RNA (ug)	small RNA enrichment	Labeling Kit	Dye	Agitation	# of replicates
AGL	one color	Human miRNA V2 Oligo Microarray	10.1	723	0.1	no	Agilent	Cy3	yes	3
AMB	one color	mirVana miRNA Bioarray V9.2	9.2	312	20	yes	Ambion	Cy5	no	3
EXQ	one color	miRCURY LNA microRNA Array v.10.0	10.0	704	1	no	Exiqon	Hy5	no	3
IVG	two color	Ncode Human miRNA Microarray V3	10.0	699	5	no	Invitrogen	Alexa5 Alexa3	no	3×2
TRY	one color	3D-Gene Human miRNA oligo chip	10.1	723	0.5	no	Exiqon	Hy5	yes	3

Agilent and Toray used its own method or software to generate a quantitative signal value and a qualitative detection call for each probe on the microarray, whereas Ambion, Exiqon, and Invitrogen did not specify the scanner or software to quantify the signals of probes in the manufacturer's protocol booklet. To generate a qualitative call for probes, we asked the technical support centers of Ambion, Exiqon, and Invitrogen about the method of detection call. We followed the methods recommended by their technical support center.

### Probe mapping

The MAQC project had a probe mapping problem in that each gene was detected by a differently designed probe between the different microarray platforms [6]. In contrast to the MAQC project, this cross-platform study of microRNA microarray has much less variability of probe mapping, because of the short length (18–23 nucleotides) of microRNAs. Instead of this probe mapping problem, we faced a different kind of annotation problem, due to the database version. The frequent update of the miRBase microRNA database [16] causes the situation that different microRNA platforms were designed based on A different version of miRBase database. Between the versions, names of some microRNAs were changed, and the sequence of some microRNAs bearing the same names were slightly changed in length. Therefore, we compared the sequences in the annotation list provided by the manufacturers. The 309 microRNAs which had the complete identical sequences probed in all different platforms were included in this study to simplify the inter-platform comparison and to avoid a bias based on miRBase version.

### Distribution profile of microRNA microarray data

It will be important to know whether all data follows a specific distribution, e.g. Gaussian or not. Thus, we checked the distribution profile of data used in this study (Figure S1 and Table S1). MicroRNA microarray data have various distribution profiles between different platforms, although microarray data tend to have positive skewness (a right-side longer tail). It has been reported that the number of genes that are expressed at a similar level is approximately exponentially distributed in typical biological samples [17]. However, the skewness and kurtosis of microRNA microarray data were far smaller than those of the exponential distribution (skewness = 4, and kurtosis = 9) (Table S1). We also checked whether non-zero log2 data were normally distributed, or not. However, non-zero log2 data did not fit to normal distribution (Figure S1B). On the other hand, the log-ratio data between two samples were approximately normally distributed (Figure S1C).

### Intra-platform data repeatability

**Figure 2:**
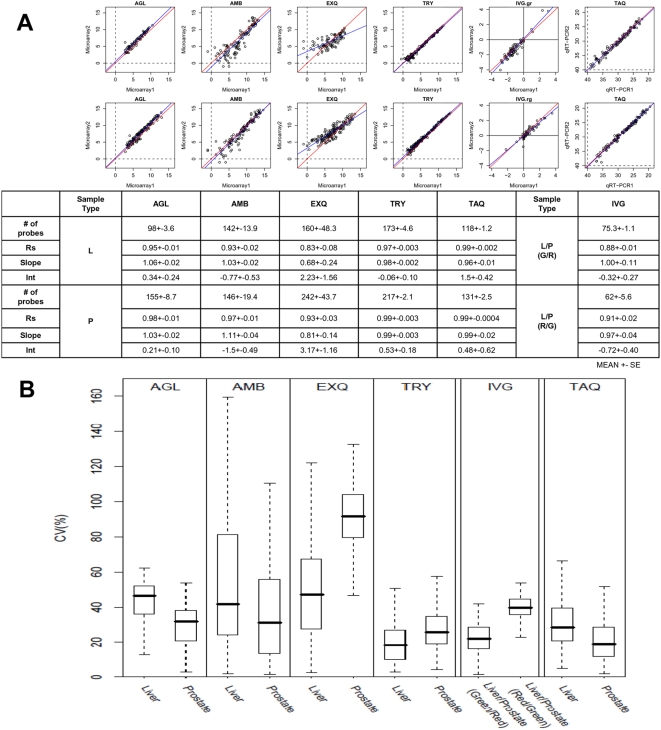
Intra-platform repeatability of quantitative assessment of microRNA expression. MicroRNA measurement of the same sample (L: human liver, P: human prostate) was replicated three times. Only data of microRNAs with positive detection call were used for analysis. 2A: Scatter plots show the correlation between replicate 1 and 2 (scatter plots for all possible combinations between three replicates were shown in Figure S4). Spearman's correlation coefficients (Rs) for replicates 1 vs. 2, 1 vs. 3, and 2 vs. 3 were calculated and summarized in lower table. Rs for the prostate sample were generally better than those for the liver sample (p = 0.0005, paired T-test). This finding suggests that repeatability of microRNA would depend on the sample cell type, and that repeatability in the case of samples expressing A higher amount of microRNAs would be better. TAQ (Taqman analysis) obtained the best Rs values despite a slightly wider spread of data. It might be a result from wider range of microRNA detection (microarray: 2^16^, Taqman: about 2^20^). 2B: Box plot of coefficient of variation (CV) for microRNA detection platforms. The coefficient of variation for each microRNA assessment was calculated by a formula, CV = (standard deviation/mean)×100, and the distribution of CV was plotted in the box plot diagram. Bold line: median, bottom and top line of the box: first and third quantile, respectively.

We examined microarray data for consistency within each platform by reviewing the repeatability at two levels: the quantitative signal values and the qualitative microRNA list agreement. To assess the data consistency of quantitative signal values, rank-correlation analysis and coefficient of variation (CV) analysis were performed. In this analysis, only data of microRNAs with positive detection call were used. Representative scatter plots of microarray platforms and the Taqman system are displayed in Figure 2A (scatter plots for all possible combinations between three replicates were shown in Figure S4). The Spearman's correlation coefficients (Rs), and the coefficient of variation (CV) between the three replicates was calculated using the 309 common microRNAs. Different platforms had various ranges of Rs values (liver: 0.82-0.96, prostate: 0.89-0.99, respectively). Thus, the 2-sample t-test and Mann-Whitney did not detect any significant difference between liver and prostate using whole data sets. However, the Rs values for prostate samples were constantly better than those for liver samples (Paired t-test: p = 0.0013, and Wilcoxon's signed-rank test: *p* = 0.0005). It is reasonable that Rs values of liver were lower than those of prostate, because higher signals in microarray data tend to have smaller data variability in general.

The distribution of CV for each platform was displayed in Figure 2B. Two platforms (AMB and EXQ) have low stringent criteria for detection call, in that all microRNAs with positive signal values after subtraction of background are considered as detected. It is also reasonable that these two platforms have higher CV values (both t-test and Mann-Whitney test: *p*<0.0001), because these platforms include microRNAs with near-zero values. In addition, the CV values of microRNA microarray platforms ranged in equivalent level to those of the Taqman assay.

**Figure 3:**
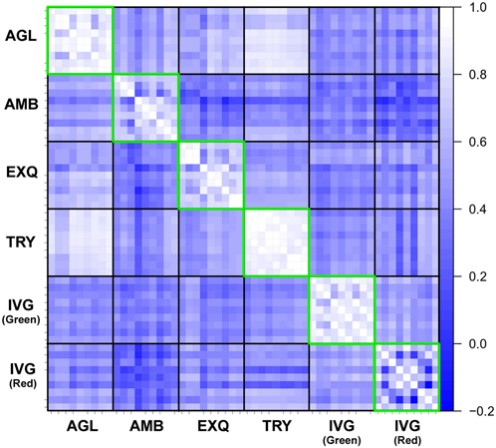
Rank correlation of log-ratios between intra- and inter-platform replications. For each platform, microRNA expression profiles in the liver and the prostate were measured three times by independent microarray chips. Therefore, 9 ( = 3×3) combinations of log-ratios (liver/prostate) for each microRNA was calculated. Then, 81 ( = 9×9) Spearman's correlation coefficients (Rs) values were calculated, and visualized in blue-white heat map. White indicates high correlation, whereas blue means low correlation. Heatmaps by Pearson's and Kendall's correlation coefficients were available in Figure S4.

Next, we assessed the variation in log-ratio measurement. For each platform, we performed triplicate experiments using human liver and prostate samples. Thus, we can generate 9 ( = 3×3) log-ratios (prostate/liver) for each microRNA. Then, we calculated the Spearman's correlation coefficients (Rs) between 9 sets of log-ratios for the detected microRNAs, and visualized these Rs values inside of green squares in a blue-white heat map (Figure 3). The means and 95% confidence intervals (95%CI) of Rs values were listed in Table S2. The Rs values were high and consistent in two platforms (AGL, and TRY), in which protocol hybridization were performed with agitation. Another reason for the inconsistency of log-ratio values in AMB and EXQ might be the low stringent criteria of detection call, which included microRNAs with near-zero values.

**Figure 4:**
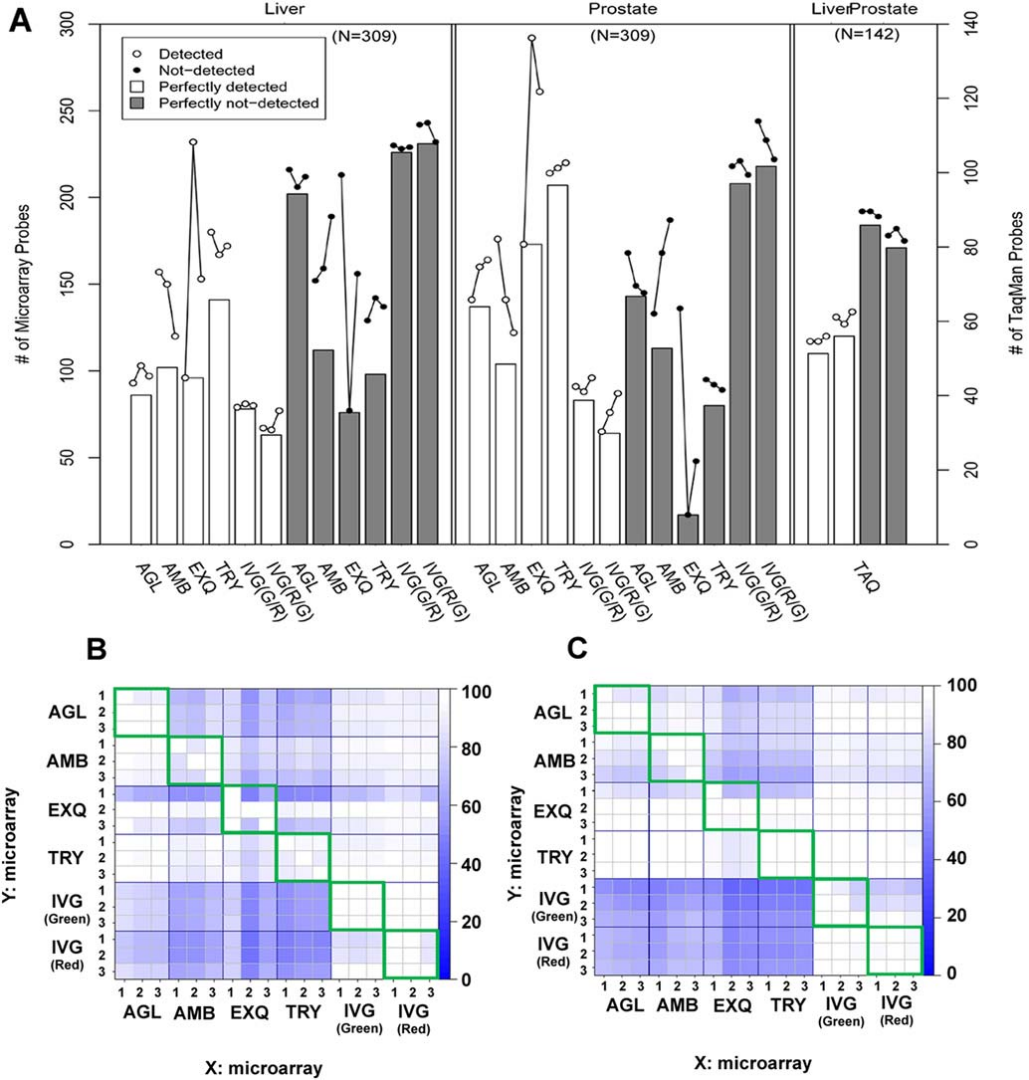
Repeatability and agreement of detection call. As a qualitative assessment of microRNA, A list of detected microRNAs should agree between different platforms. Detection call of microRNA for each platform was performed according to different criteria recommended by the manufacturer. 4A: The number of detected microRNAs. Closed circles: detected microRNAs, open circles: not detected microRNAs, white bar: perfectly detected microRNAs, which were detected in all three replications, gray bar: perfectly not-detected microRNAs, which were not detected in all three replications. For the Taqman analysis, amplified microRNAs within 40 cycles were considered as detected. 4B & C: Agreement rate of detection call list between intra- (inside of green squares) and inter-platform (outside of green squares) replications using liver (4B) and prostate (4C) samples. The percent agreement of detected microRNAs was calculated as the number of microRNAs detected by platform Y relative to the number of microRNAs detected by platform X. Therefore, two blocks in A diagonally symmetric position are not always the same color, because the denominators are different.

To assess variation in the qualitative measures, the percentage of 309 microRNAs with concordant detection calls between replicates of the same sample type was calculated on each platform (line graphs in Figure 4A). As expected, microarray signals from liver samples were generally weaker than those of prostate samples (Figure 1). Thus, the percent of detected microRNA subset in liver samples was significantly smaller than that in prostate samples (Figure 4A, paired T-test: *p* = 0.0003, and Wilcoxon's signed rank test: *p* = 0.0005). In the current study, we used criteria of detection call of microRNAs that the manufacturers recommended. However, the stringency of these detection call criteria was very different. For AMB and EXQ array, all microRNAs with positive signal were handled as detected microRNAs, whereas other manufacturers provided their own formula as detection call criteria. This difference in the detection call stringency may result in the divergence of detected microRNA percentage. Thus, detected microRNA percentage of AMB and EXQ array were less stable in three replicates (t-test and Mann-Whitney test of standard deviation, *p* = 0.0011 and 0.004, respectively) than the others.

**Figure 5:**
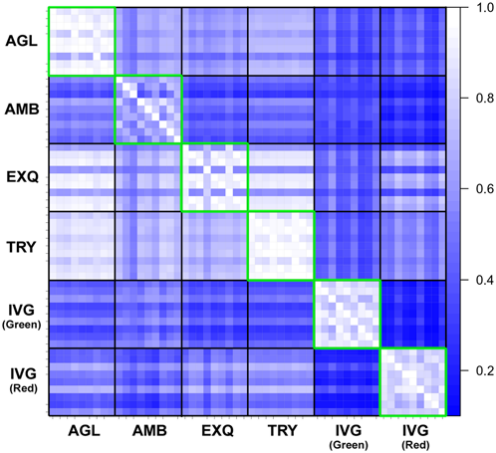
Agreement in the list of differentially expressed microRNAs. This graph indicates the concordance of microRNAs identified as differentially expressed for pairs of platforms, labeled as X and Y. A list of differentially expressed microRNAs between human liver *vs.* human prostate was generated for each platform (using the 309 common microRNAs with ≥two-fold change) and compared for commonality to other platforms. No filtering related to the qualitative detection call was performed. The color of the square in the matrix reflects the percent overlap of microRNAs on the list for the platform X (listed in column) that are also present on the list for the platform Y (listed in row). A light-colored square indicates a high percent overlap between the microRNA lists at both platforms. A dark-colored square indicates a low percent overlap, suggesting that most microRNAs identified in platform X were not identified in platform Y. Note: the graph is asymmetric and not complementary, for the same reason as in Figure 4B and C.

Intra-platform concordance in detected microRNA list was shown inside of green squares in Figure 4B and 4C. It is reasonable that AMB and EXQ with instable percentage of detected microRNAs also had higher inconsistency in the detected microRNA list than the others. Intra-platform concordance in a list of differentially expressed microRNAs was illustrated inside of green squares in Figure 5. The means and 95%CIs of agreement percentages were listed in Table S3. AGL and TRY had more than 90% concordance of differentially expressed microRNAs list within intra-platform replicates.

### Inter-platform data comparability

MicroRNA expression values generated on different platforms cannot be directly compared because unique labeling methods and probe sequences will result in variable signal distributions for probes that hybridize to the same target microRNAs. (Figure S1) Alternatively, the relative expression between a pair of sample types should be maintained across platforms. For this reason, we examined the microarray data for comparability between platforms by reviewing liver sample to prostate sample expression values with two different levels: rank correlation of the log-ratio as qualitative assessment, and the microRNA list agreement (detection call and identification of differentially expressed microRNAs) as qualitative assessment.

To show the inter-platform concordance in the detected microRNA list, the percentage of 309 microRNAs with concordant detection calls between replicates on different platforms was calculated and visualized outside of green squares in Figure 4B and 4C. The median percentages of inter-platform detection concordance were 74.0% and 72.1% for liver and prostate sample, respectively. There was no statistical difference in detection call concordance between liver and prostate samples. For both samples, these percentages were widely distributed, ranging 56.3–97.9% and 58.2–95.9%, respectively, because the difference in detection call stringency lead to a divergence in detection call rate across the platforms.

The comparability of results across the platforms was also examined using a rank correlation metric. For rank correlation, only detected microRNAs from the common 309 gene list were included in the analysis. Log-ratios for the differential expression observed between liver sample replicates and prostate sample replicates were calculated for the generally detected common microRNAs and then compared across the platforms. The rank correlations of the log-ratios are displayed visually in Figure 5A. Good agreement was not observed between the platforms, compared to the original MAQC report. In fact, the best correlation was obtained between AGL and TRY (Rs = 0.8717), and the median rank correlation was 0.55 between the microarray platforms.

**Figure 6:**
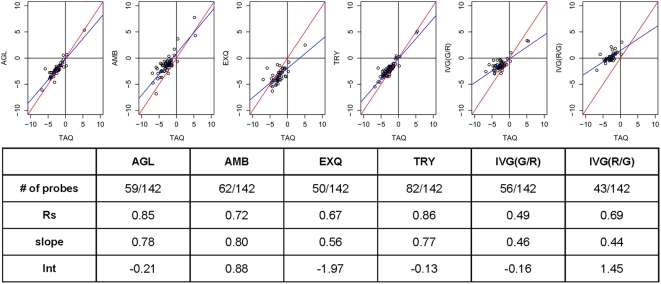
Correlation between microarray and Taqman data. The scatter plots compare the log-ratio differential expression values from each microarray platform relative to values obtained by the Taqman assays. Each point represents a microRNA that was measured on both the microarray and Taqman assays. Only microRNAs that were generally detected in both human liver and prostate were used in the comparisons. Among 142 total microRNAs assayed by the Taqman system, the number of microRNAs analyzed for correlation to the Taqman assays are listed in the table. The red and blue lines shown are the ideal Y = X line, and the regressed line from the scatter plots, respectively. Spearman's correlation coefficients (Rs), slope and Y intercept of regressed line were shown in the table.

For the list overlap of differentially expressed microRNAs, all 309 common genes were considered. A list of differentially expressed microRNAs was generated for each platform and compared to lists from the other platform. A percent score was calculated to indicate the number of microRNAs in common between each pair of platforms. The percentage of overlap for each comparison is displayed in Figure 5. Note the graphic comparisons are asymmetrical indicating the analysis is performed in two directions. That is, the percentage of platform Y microRNAs on the list from platform X can be different from the percentage of platform X microRNAs on the platform Y list. In contrast with one color platforms, IVG (two-color method) identified A much lower number of differentially expressed microRNAs, probably due to log-ratio compression (Figure 6). Therefore, percentages of list overlap between IVG and one-color platforms were generally low. AGL, EXQ and TRY had a good concordance in terms of identifying differentially expressed microRNAs.

### Correlation to Taqman assay

In the MAQC project, the quantitative accuracy of several non-microarray devices was checked, then quantitative RT-PCR (Taqman system) was selected as a validation method of microarray data. In the microRNA research field, several different types of quantitative RT-PCR (qRT-PCR) methods are in use, such as qRT-PCR using stem-loop shaped RT-primer, Taqman system, Applied Biosystems) [18], qRT-PCR using locked nucleic acid primers (Exiqon) [19], and qRT-PCR with poly-A tailing (QIAGEN, Stratagene). In this study, we also used the Taqman microRNA assay system as a validation method, which is a method most widely used. Further comparisons between each microarray platform relative to the TaqMan assays are presented as scatter plots in Figure 6. One hundred forty two microRNAs were randomly selected from 309 common microRNAs to the microRNA platforms, then the expression levels of these 142 microRNA in the human liver and prostate were measured by Taqman system. Good correlation coefficients (Rs = 0.85,0.86) were obtained from AGL and TRY platforms, respectively. In all platforms, especially IVG (two-color method), log-ratio compression (slope<1) was observed. In the original MAQC paper, a two color method showed log-ratio compression in the comparison with Taqman assays. Thus, our finding is a consistent result.

**Figure 7:**
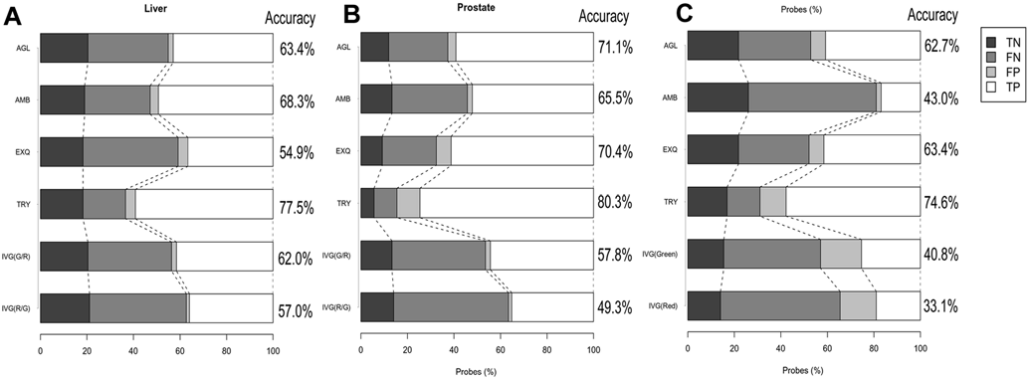
Agreement of microRNA list between microarray platforms and the Taqman assay. MicroRNAs that were listed, or not listed in both microarray and the Taqman assay, were considered as true positive (TP) or true negative (TN), respectively. MicroRNAs that were listed in either microarray or Taqman assays, were handled as false positive (FP) or false negative (FN), respectively. 7A and B: These graphs indicated the concordance of detection call between microarray platforms and the Taqman assay, using 142 microRNAs assayed by both microarray and the Taqman system. 7C: This bar graph demonstrated the concordance of microRNAs identified as differentially expressed between microarray platforms and the Taqman assay system.

Figure 7 demonstrated microRNA list agreement of detected microRNAs (Figure 7A and 7B) and differentially expressed microRNAs (Figure 7C). For detection call of microRNAs, there were few false positive and many false negative results. Thus, the microarray method is a device with high specificity and less sensitivity, compared to the Taqman assay. In identifying differentially expressed microRNAs, high concordance ratios (81.69%, 88.73%) to the Taqman assay were obtained in EXQ and TRY platforms, respectively. In contrast, IVG has very low true positive results, probably because two color method had a severe log-ratio compression.

## Discussion

The results of the current study provide information about the potential advantages and problems of microRNA microarray technologies as a tool providing microRNA expression data for research and future clinical purposes.

In the original MAQC paper, the median values of CV for gene expression microarray ranged from 5 to 20%, whereas those of CV in this study ranged from 20 to 90%, approximately. We wondered why the CV values in this study were much higher than those in the MAQC paper, although the Rs values in this study were similar to those in the MAQC papers. One possible explanation was that the data distributions of the replicated data sets were not well centered due to a lack of data normalization. For the gene expression microarray analysis, data are generally normalized under an assumption that the total amount of mRNA is constant between different samples. However, microRNA microarray data generated from the same amount of total RNA were not normalized in general, because we know that the amount of microRNA varies depending upon cell types, such as normal tissue *vs.* cancer [15]. To assess whether this explanation is true or not, we normalized the microRNA microarray data within replicates of the same samples in the same platforms (Figure S2). The CV values were drastically improved after the quantile normalization within the same replicates. The median values of CV in Figure S3 were significantly lower than those in Figure 3 (paired t-test, p = 0.03813). This finding suggests that normalizing microRNA microarray data would be beneficial to improve data repeatability and consistency in situations when the amounts of microRNAs in the samples are assumed to be constant. Furthermore, we should develop a universal method that can perform a reasonable normalization between different cell types containing different amounts of microRNAs.

This normalization problem is associated not only with microRNA microarrays, but also with the Taqman assay. Because we have not discovered reliable housekeeping microRNAs, the Taqman assay measures just Ct values without normalization, which are obtained using the same amount of total RNA. This fact may result in the relatively high CV values that are ranging in the equivalent level to microRNA microarray platforms. In other words, a similar level of repeatability would be a relative advantage of microarray platforms to the Taqman system.

Another problem with microRNA microarray platforms is a divergence in the stringency of the detection call criteria. The detection call criteria should be adjusted to each platform, in order to obtain reliable and repeatable data. However, too much divergence in the percentages of detected microRNAs would result in the disagreement in the results of further analyses, which may induce underestimated impressions and reputations of microRNA microarray technology. Therefore, this report emphasizes the necessity of a larger project that will solve specific microRNA problems, such as normalization and detection call stringency, and that builds a consensus in all aspects of the microRNA microarray analysis.

In the MAQC project, seven different platforms (Applied Biosystems, Affymetrix, Agilent, Eppendolf, GE Healthcare, Illumina, and NCI array) were compared [6]. In the current study, five platforms (Agilent, Ambion, Exiqon, Toray, and Invitrogen) were studied. Thus, only one company (Agilent) was overlapped. This fact indicates that the tips developed in gene expression microarray field are not inherited well into microRNA microarray. One example is the hybridization method. Currently, many gene expression microarray platforms employ a dynamic hybridization method to generate repeatable and reproducible data. In the current study, microRNA microarray platforms with dynamic hybridization systems (AGL and TRY) showed relatively better results than those with static hybridization systems. It is easily imaginable that the addition of agitation into the hybridization procedures of AMB, EXQ, and IVG platforms would improve data quality. In the microRNA research field, Luminex Corp. (Austin, TX) provides a beads-hybridization-based microRNA detection system (FlexmiR). The beads-hybridization is one form of the dynamic hybridization methods, and has high sequence specificity [15]. However, this system can detect microRNAs only in the miRBase version 8 list, when we performed the experiments. Therefore, we excluded this system from the current study.

The updated version of the miRBase list will include newly registered microRNAs. These newly added microRNAs are expected to be expressed at relatively low levels. Therefore, adding new microRNAs with low expression would cause poorer performance in repeatability or reproducibility, even when using the same platforms. It suggests that a standard set of microRNAs would be needed to compare the performances between microRNA microarray platforms designed according to different miRBase versions.

In this study, we assessed the repeatability and comparability of microRNA microarray among several commercially available platforms. Different from mRNA expression microarray, microRNA microarray requires another important characteristic in the assay. For mRNA microarray, the probe(s) for each gene can be designed at the unique DNA sequence site on the gene to avoid the cross-hybridization. However, the short length of microRNAs (18–23 nucleotides) restricts the flexibility of probe design. Moreover, there are many same-family microRNAs which have high sequence homology. Thus, microRNA microarray requires an ability to distinguish these same-family microRNAs with high specificity. In this study, we did not assess the sequence specificity of the microRNA microarray. This issue should be addressed in future studies.

Currently, the MAQC project is on-going in the second phase, and the US FDA released the second version of draft guidelines for IVDMIA in July, 2007 (http://www.fda.gov/cdrh/oivd/guidance/1610.pdf). Actually, several microarray platforms for mRNA expression have already been approved and utilized in the clinical field as IVDMIAs, e.c., MammaPrint® for breast cancer, and Pathwork® Tissue of Origin Test for unknown origin tumor. Regarding the microRNA microarray, this study demonstrated that some platforms of microRNA microarray have intra-platform repeatability as high as that of the mRNA expression microarray demonstrated in the MAQC papers. Thus, our finding indicated that the microRNA microarray may have high potential as a clinical diagnostic tool when good diagnostic microRNA markers are available. To date, at the research level, many papers have described the physiological and pathological significance of microRNAs, and reported potential biomarker microRNAs. However, the reproducibility of the microRNA microarray has not been assessed by a multi-center study, such as the MAQC project. Furthermore, there are some microRNA-specific problems to be solved, such as building consensus on normalization of the microRNA expression data and the specificity of microRNA detection to distinguish microRNAs with high sequence homology. Thus, a large-scale multi-center quality control project specific to the microRNA microarray is required before its clinical application.

In a review article, Shendure, described that the next-generation high throughput sequencer would replace DNA microarray technology in the transcriptome research field [20]. The next-generation sequencing technology has been applied to microRNA detection [21]. Is this the beginning of the end of the microRNA microarray? As far as we know, the current report is the first paper to compare the several platforms of microRNA microarrays regarding their performances. We have not fully evaluated the advantages and disadvantages of microRNA microarray yet. Therefore, it is too early to answer this question and it should be addressed in a near future study.

In conclusion, this study demonstrated that the microRNA microarray has high intra-platform repeatability and comparability to quantitative RT-PCR of microRNA. However, the current lineup of commercially available microRNA microarray systems fails to show good inter-platform concordance, probably because of severe divergence in stringency of detection call criteria between different platforms. This study provided the basic information about the performance and the problems specific to the current microRNA microarray systems.

## Materials and Methods

### MicroRNA microarray platforms

Five commercially available microRNA microarray platforms; Agilent Technologies (Santa Clara, CA), Ambion (Austin, TX), Exiqon (Vedbaek, Denmark), Invitrogen (Carlsbad, CA), and Toray (Tokyo, Japan) were tested in this study. In all assays, we performed microRNA microarray assays according to the manufacturer's protocols available in April, 2008.

### RNA samples

Our preliminary experiments showed that the amount of microRNA obtained from the same amount of total RNA depends on the tissue types of the samples (data not shown). This finding suggested that reproducibility or detection call rate of microRNA microarray analysis might vary depending on the amount of microRNA contained in the total RNA. To assess the reproducibility of microRNA microarray data using the different tissue types, we chose both tissue samples, which contain relatively small and large amount of microRNA. Our preliminary data shows that mouse liver tissue contains A relatively small amount of microRNAs. Therefore, we used two types of total RNA, FirstChoice® Human Liver Total RNA (Ambion, lot no. 040000129) and FirstChoice® Human Prostate Total RNA (Ambion, lot no. 050500710), in this study. As shown in Figure 1, microRNA expression level in human liver was lower than in human prostate. For Ambion's microRNA microarray, small RNA fractions purified from these total RNAs were used. For other microarrays, total RNAs were directly processed. The amounts of total RNA used for the assays were decided according to the manufacturer's protocols (Table 1). The platform-specific external controls were added to the samples prior to labeling for all platforms.

### Labeling and hybridization

For the Invitrogen microarray, RNA samples were labeled using a two-color and dye-swapping protocol. For other microarrays, a one-color protocol was used. Three replicate assays for each sample were independently processed. In the two-color protocol, two RNA samples differently labeled by Alexa 532 and Alexa 645 were simultaneously hybridized on the same microarray chip. In addition, to normalize the dye-specific bias, RNA samples were labeled by switched dye combination. The microarray data from these two sets of two color scanning image were integrated. On the other hand, in a one-color protocol, each RNA sample was labeled using a single dye, and two RNA samples were hybridized separately on two microarray chips. All target labeling and hybridizations were performed in triplicate, according to the manufacturer's protocols. (Notes: A recent Exiqon protocol utilizing agitated hybridization was not provided in April 2008, when our experiments were performed)

### Microarray chip scanning

#### (1) Agilent microarray

Microarray slides were scanned using an Agilent microarray scanner G2505B (Agilent technology) and microarray images were automatically analyzed using Feature extraction™ software, version 9.5.1.1 (Agilent technology). In this study, the gTotalGeneSignal values were used as the feature intensities, according to the procedures recommended by Agilent.

#### (2) Ambion microarray

Microarray slides were scanned USING a ProScanArray™ microarray scanner (PerkinElmer Inc. Waltham, MA). For each scanning, a photomultiplier setting of the red channel was manually adjusted to 55, and the obtained microarray images were analyzed using the Genepix Pro™ 4.0 software (Molecular Device, Sunnyvale, CA). Spots that might be associated with artifacts were eliminated using software- and visual-guided flags. In this study, the median values of the foreground signal minus the local background were represented as feature intensities.

#### (3) Exiqon microarray

Microarray slides were scanned using Agilent microarray scanner G2505B and the obtained microarray images were analyzed using the Genepix Pro™ 4.0 software. Artifact-associated spots were eliminated both by software- and visual-guided flags. In this study, the median values of the foreground signal minus the local background were represented as feature intensities.

#### (4) Invitrogen microarray

Microarray slides were scanned using Agilent microarray scanner G2505B and the microarray image was analyzed using the Genepix Pro™ 4.0 software. Spots that might be associated with artifacts were eliminated using by software- and visual-guided flags. In this study, the median values of the foreground signal minus the local background were represented as feature intensities.

#### (5) Toray microarray

Microarray slides were scanned using ProScanArray™ microarray scanner where the photomultiplier settings of the red channel were manually adjusted according to the procedures recommended by the manufacturer. Each microarray was scanned three times, then merged into one data, and the merged data were analyzed using the Genepix Pro™ 4.0 software. Spots that might be associated with artifacts were eliminated using software- and visual-guided flags. In this study, the median values of the foreground signal minus the local background were represented as feature intensities.

### Microarray data processing

In ordinary mRNA expression microarray, it is a standard data processing procedure to normalize the microarray data with an assumption that the whole mRNA expression signal is constant among the samples. However, in the microRNA analysis, the amount of microRNA contained in the same amount of total RNA varied depending on the tissue or cell types. All microarray data were registered into NCBI's Gene Expression Omnibus (GEO) database (http://www.ncbi.nlm.nih.gov/projects/geo/). The accession numbers ARE listed in Table S4 of Supporting information.

### The detection call criteria

Detection call criteria for Agilent, Toray, and Invitrogen were described in the manufacturers' protocol handbook, whereas those for Ambion and Exiqon were not available. Thus, we asked customer support offices of Ambion and Exiqon about their recommended detection call criteria (contacted in May–June, 2008). For the Agilent array, gIsGeneDetected values in output data sheet were used for detection call. For Ambion and Exiqon arrays, customer service offices of both manufacturers recommended handling all spots with over 0 intensities as detected spots. For the Invitrogen array, the lower limit of detection is eight times the median local background of all array features. For the Toray array, positive detection call was defined as spots in which signal intensities showed greater than the upper limits of 95% confidence interval of all blank spots' signal intensities.

### Real-time quantitative PCR for microRNA expression

To validate the microRNA expression in each sample, we measured the expression of 171 microRNAs by using a qRT-PCR platform: TaqMan microRNA Assays (Applied Biosystems Inc.) and ABI 7300 Sequence Detector™. This qRT-PCR method detects specifically mature microRNAs, but not precursor microRNAs. To perform this TaqMan assay, we used the same amount of total RNA, and Ct-values were recorded. Then, the value of 2^(40-Ct)^ represents the expression level of the target microRNA.

### Probe mapping

The probe annotations for all microarray platforms and qRT-PCR were provided by the manufacturer. The official annotation of microRNAs in the miRBase Database (http://microrna.sanger.ac.uk/) is being updated frequently. The release version of the official database was 11.0 when this study started. However, the microRNA annotation version used for the microarray probe design was different among the five different microarray platforms in April 2008. Agilent and Toray are based on the Sanger miRBase Database, release 10.1 and 11.0, respectively. Exiqon and Invitrogen are based on the release 10.0, and Ambion is based on the release 9.2. To analyze the different formats of microarray data, we extracted microRNA expression data exactly matched to release 10.1. To compare the microRNA profile between two different microarray platforms, we used all overlapped microRNAs available in both microarray platforms. The number of human microRNAs common among all five microarrays was 310. To validate microRNA microarray data, randomly selected 146 human microRNAs were measured by the Taqman qRT-PCR system.

### Signal repeatability and reproducibility

To assess signal repeatability and reproducibility of each microarray platform, we utilized the methods that the MAQC Project used [6], such as calculating the Spearman's correlation coefficient (Rs), and the coefficient of variation (CV) of the signal or Cy3/Cy5 values for one or two color method, respectively. The CV for each microRNA assessment was calculated by a formula, CV = (standard deviation/mean)×100.

### MicroRNA list agreement

A list of detected microRNAs in each sample and the differentially expressed microRNAs between two samples were identified for each assay. The criteria of differential expression were that a difference of two microRNA is greater than two-fold. The percent agreement of microRNAs was calculated as the number of microRNAs detected by platform Y relative to the number of microRNAs detected by platform X. For the percent agreement of differentially expressed microRNAs, the 95% confidence interval of percent agreement between platforms was estimated from distribution of percentages calculated from 81 ( = 9×9) possible combination of data sets (Figure 5 and Table S3).

### Log ratio comparability

To compare the similarity of the log ratio for each microRNA between each microarray platform, we determined the slope and intercept of the orthogonal regression between pairs of the log ratio in each microarray platform. The log ratio of each microRNA was calculated as the average of log signals in the liver sample minus the average of log signals in the prostate sample. The slope and intercept are determined by the formula y = ax+b, where “y” is the log ratio from platform Y, “x” is the log ratio from platform X, and the ideal slope is 1. For the slope, the difference from the ideal slope (a = 1) indicates the compression or expansion of the log ratios in one platform relative to the other. For the intercept, the distance of zero means the platform-specific bias between two microarray platforms.

Comparability between a pair of each platform was also examined using Spearman's rank correlations of the log ratios. This value compares the relative position of a microRNA in the platform X rank order of the log ratio (fold change) values against its position in the platform Y rank order.

### Concordance with qRT-PCR

The percentage of overlapping microRNAs between each microarray platform and qRT-PCR was a measure of the reproducibility of lists of differentially expressed microRNAs. We considered that the agreement of detected microRNAs in each sample and differentially expressed microRNAs between two samples for each microarray platform. For each platform, microRNA expression profiles in the liver and the prostate were measured three times by independent microarray chips. Therefore, 9 ( = 3×3) combinations of log-ratios (liver/prostate) for each microRNA was calculated. The microRNAs that had consistently greater than or equal to two-fold difference in these 9 ratios were assigned as differentially expressed microRNAs for each platform. Because we considered that qRT-PCR was true, true positive (TP) was detected in both of microarray and qRT-PCR, true negative (TN) was not-detected in both of microarray and qRT-PCR, false positive (FP) was only detected in microarray, and false negative (FN) was only detected in qRT-PCR. The formula for accuracy is (TP+TN)/(TP+TN+FP+FN).

## Supporting Information

Figure S1 Distribution profile of microRNA microarray data Histograms of microRNA microarray data. All 309 microRNA data of each microarray platform were plotted in a histogram. In addition, 142 microRNA data of Taqman RT-PCR data were displayed in the same format. Negative log2 values were handled as 0 (0 = log_2_1).
(4.66 MB TIF)

Figure S2 Probability plots of microRNA microarray data distribution. To show the normality of the distribution of non-zero data, The probability plot of each data set was generated using non-zero log2 values, excluding 2.5% of values in both sides. If distribution of the data is normal, this probability plot would be a line. In most of cases, kurtosis of the data distribution was around 2.
(8.06 MB TIF)

Figure S3 Probability plots of the distribution of log-ratio values. To demonstrate the normality of the distribution of log-ratio values, probability plots of log-ratio data were generated using 95% of middle log-ratio data. Lilliefor's test showed that the null hypothesis was not rejected in EXQ and TRY, which means that the distribution of log-ratio data in EXQ and TRY array were quite similar to normal distribution. p: p-values of Lilliefor's test.
(3.62 MB TIF)

Figure S4 Scatter plots showing correlations between the same replicates. Red and blue lines indicate the ideal Y = X line, and linear regressed line of scattered dots. S1A∼D: For one-color platforms, representative signal values of microRNA were plotted. S1E: For two-color platform (Invitrogen), log2-ratios (liver/prostate) of microRNA were plotted.
(9.50 MB TIF)

Figure S5 Effect of normalization on the rank-correlation of microRNA microarray. At first, we performed quantile normalization within the same replicates using one-color platform data. Then, the Spearman's correlation coefficients (Rs) were calculated. Because the quantile normalization changes values of microRNAs but not rank of microRNAs, Rs values in Figure 2 and Figure S3 were the same.
(8.12 MB TIF)

Figure S6 Effect of normalization on the coefficient of variation In contrast to Spearman's correlation coefficients in Figure S3, the coefficients of variation (CV) were drastically improved after the quantile normalization within the same replicates. The median values of CV in Figure S3 were significantly lower than those in Figure 3 (paired t-test, p = 0.03813). The CV of AGL and TRY were within the range of CV demonstrated in the original MAQC project paper.
(5.07 MB TIF)

Figure S7 Correlation of log-ratios between intra- and inter-platform replications. Heatmaps of Pearson's correlation coefficients and Kendall's rank correlation coefficients. Both heatmaps had a similar pattern to heatmaps using Spearman's correlation coefficients in Figure 3.
(6.08 MB TIF)

Table S1 Skewness and Kurtosis of microRNA microarray data distribution Skewness and kurtosis of each data set was calculated using all expression data or non-zero log2 data of 309 microRNAs. A symmetric distribution has 0 skewness. A distribution with positive skew has a longer right tail, while a distribution with negative skew has a longer left tail. The kurtosis of the normal distribution is 3. A high kurtosis distribution has a sharper peak and longer, fatter tails, while a low kurtosis distribution has a more rounded peak and shorter thinner tails. This table demonstrated that microRNA microarray data tend to have a positive skewness.
(0.03 MB DOC)

Table S2 Rank correlation coefficients of log-ratios between intra- and inter-platforms of microRNA microarray. For rank correlation calculation, we used data of detected microRNAs that meet the detection criteria of each manufacturer. Both prostate and liver samples have triplicated data sets. Thus, 9 ( = 3×3) sets of log-ratios (prostate/liver) of microRNAs were generated. For intra-platform correlation, rank correlation coefficients of 36 ( = 9×8÷2) combinations were calculated, whereas, 81 ( = 9×9) coefficients were calculated for inter-platform correlation. Upper values: Spearman's correlation coefficients, Lower values: 95% confidence intervals.
(0.03 MB DOC)

Table S3 List agreement of differentially expressed microRNA This table showed percentage of concordance in detecting differentially expressed microRNAs. The values in the upper portion of cells reflects the mean percent overlap of microRNAs on the list for the platform X (listed in column) that are also present of the list for the platform Y (listed in row), whereas the values in the lower portion were 95% confidence intervals of the mean percentage. A higher value indicates a high percent overlap between the microRNA lists at both platforms. A lower value indicates a low percent overlap, suggesting that most microRNAs identified in platform X were not identified in platform Y. Therefore, the table is asymmetric and not complementary.
(0.03 MB DOC)

Table S4 Accession numbers of microarray data All microarray data were registered into NCBI's Gene Expression Omunibus (GEO) database (http://www.ncbi.nlm.nih.gov/projects/geo/). All data were available to public on March 30, 2009.
(0.04 MB DOC)
