# Supplementary figures and images for: Intra-Platform Repeatability and Inter-Platform Comparability of MicroRNA Microarray Technology
Source: PLoS One. 2009 May 14;4(5):e5540. doi: 10.1371/journal.pone.0005540 (PMC2677665; doi:10.1371/journal.pone.0005540)

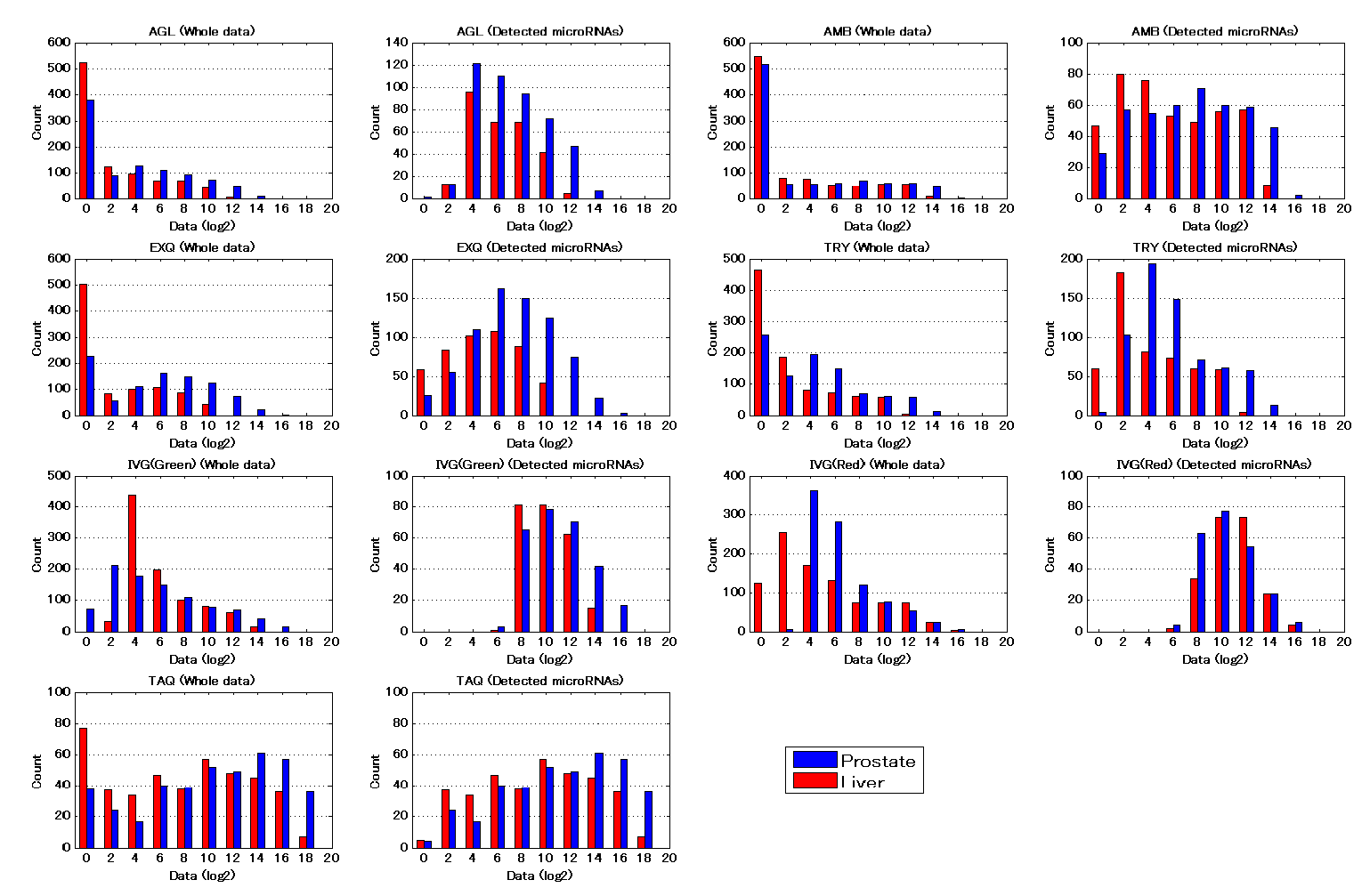

Supplement: Figure S1 — Distribution profile of microRNA microarray data Histograms of microRNA microarray data. All 309 microRNA data of each microarray platform were plotted in a histogram. In addition, 142 microRNA data of Taqman RT-PCR data were displayed in the same format. Negative log2 values were handled as 0 (0 = log21). (4.66 MB TIF) [file pone.0005540.s001.tif]

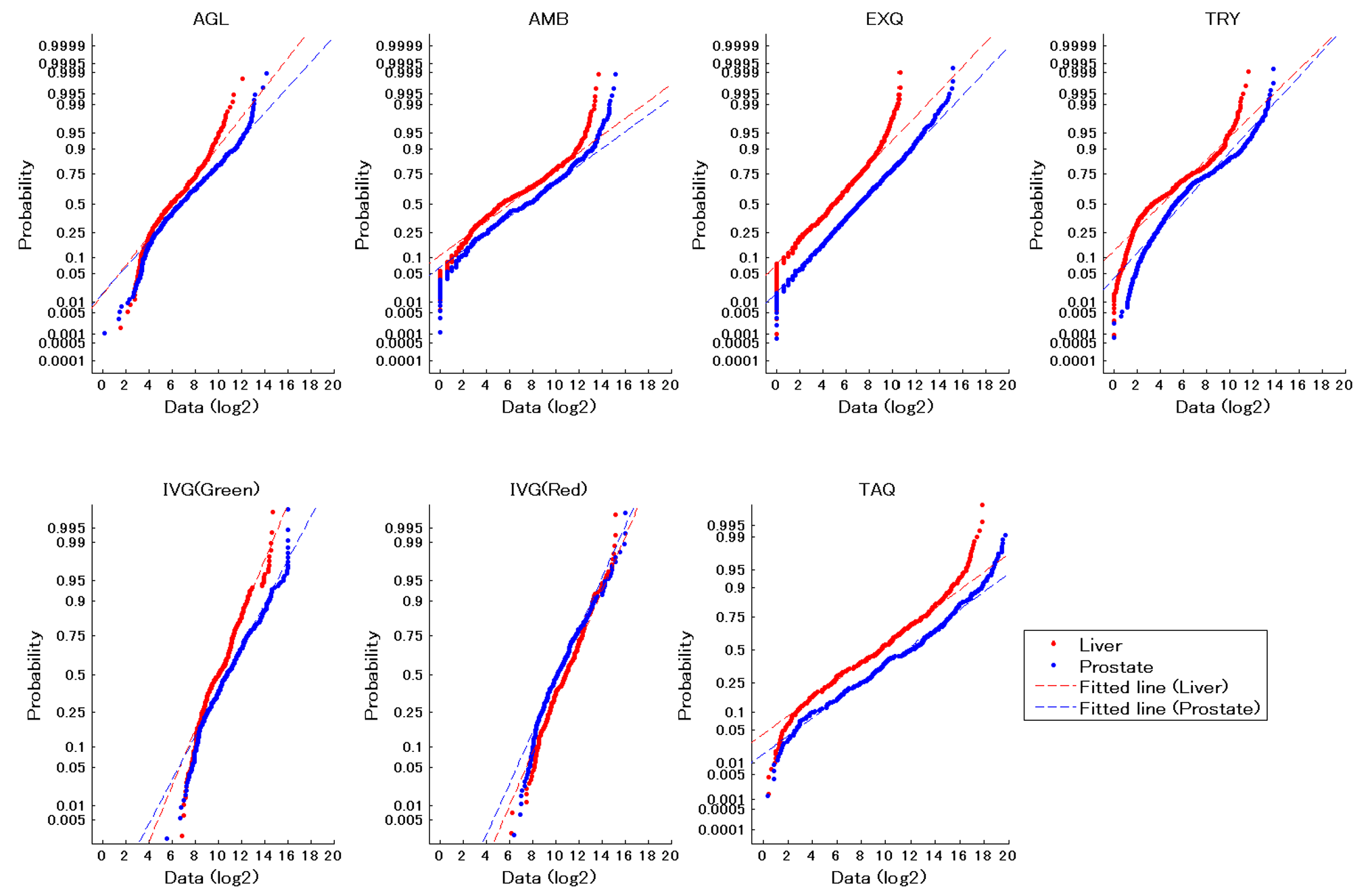

Supplement: Figure S2 — Probability plots of microRNA microarray data distribution. To show the normality of the distribution of non-zero data, The probability plot of each data set was generated using non-zero log2 values, excluding 2.5% of values in both sides. If distribution of the data is normal, this probability plot would be a line. In most of cases, kurtosis of the data distribution was around 2. (8.06 MB TIF) [file pone.0005540.s002.tif]

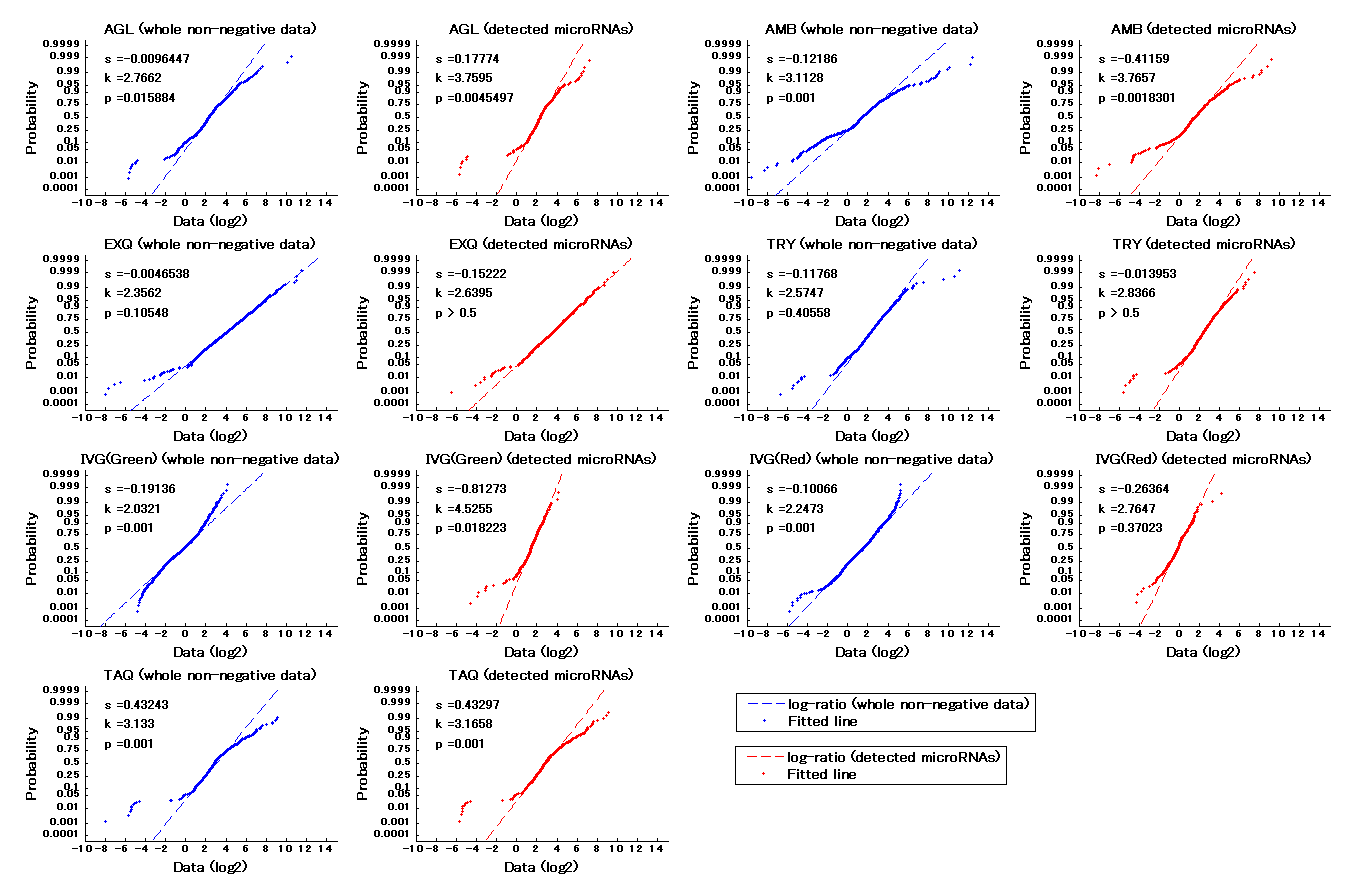

Supplement: Figure S3 — Probability plots of the distribution of log-ratio values. To demonstrate the normality of the distribution of log-ratio values, probability plots of log-ratio data were generated using 95% of middle log-ratio data. Lilliefor's test showed that the null hypothesis was not rejected in EXQ and TRY, which means that the distribution of log-ratio data in EXQ and TRY array were quite similar to normal distribution. p: p-values of Lilliefor's test. (3.62 MB TIF) [file pone.0005540.s003.tif]

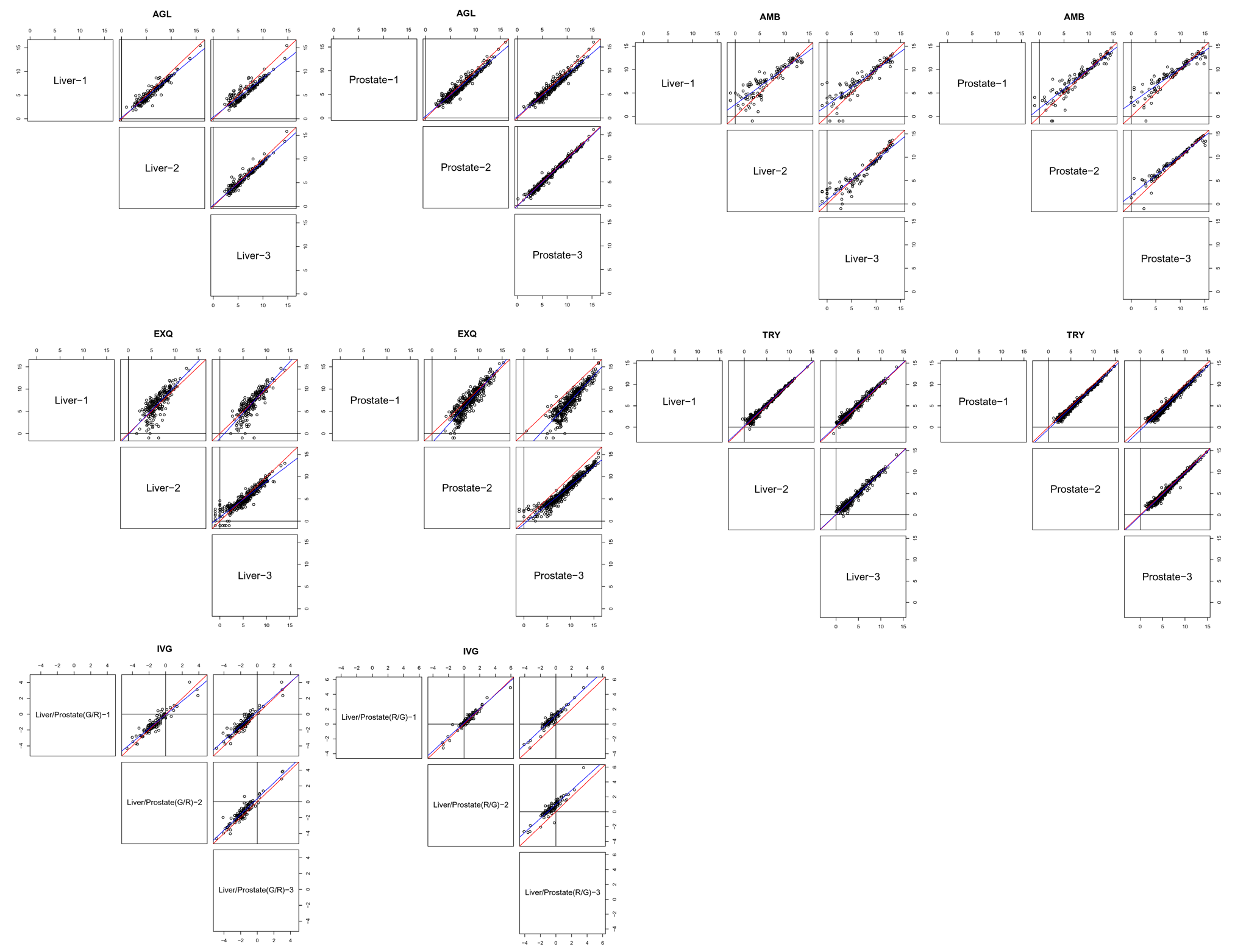

Supplement: Figure S4 — Scatter plots showing correlations between the same replicates. Red and blue lines indicate the ideal Y = X line, and linear regressed line of scattered dots. S1A∼D: For one-color platforms, representative signal values of microRNA were plotted. S1E: For two-color platform (Invitrogen), log2-ratios (liver/prostate) of microRNA were plotted. (9.50 MB TIF) [file pone.0005540.s004.tif]

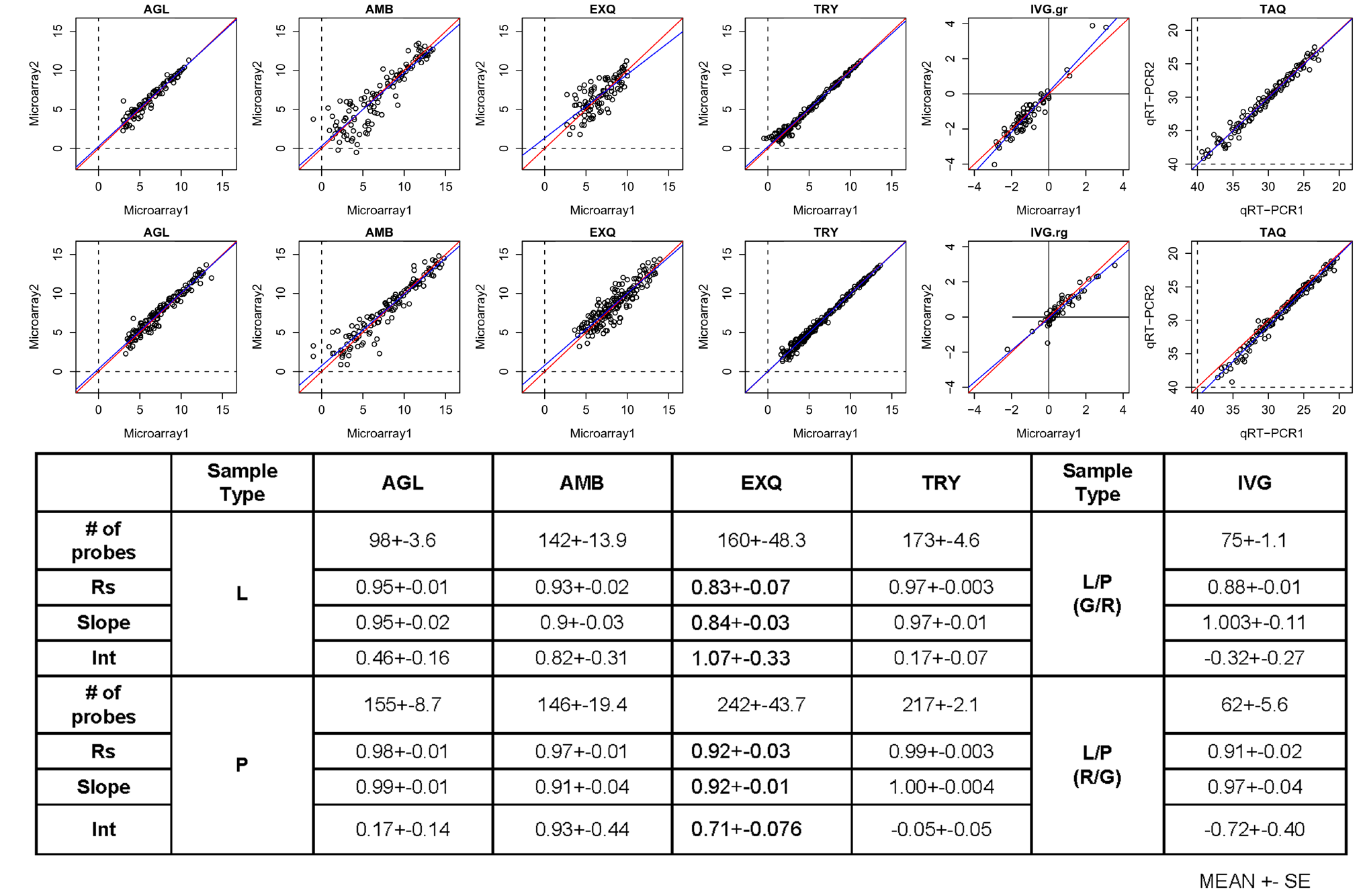

Supplement: Figure S5 — Effect of normalization on the rank-correlation of microRNA microarray. At first, we performed quantile normalization within the same replicates using one-color platform data. Then, the Spearman's correlation coefficients (Rs) were calculated. Because the quantile normalization changes values of microRNAs but not rank of microRNAs, Rs values in Figure 2 and Figure S3 were the same. (8.12 MB TIF) [file pone.0005540.s005.tif]

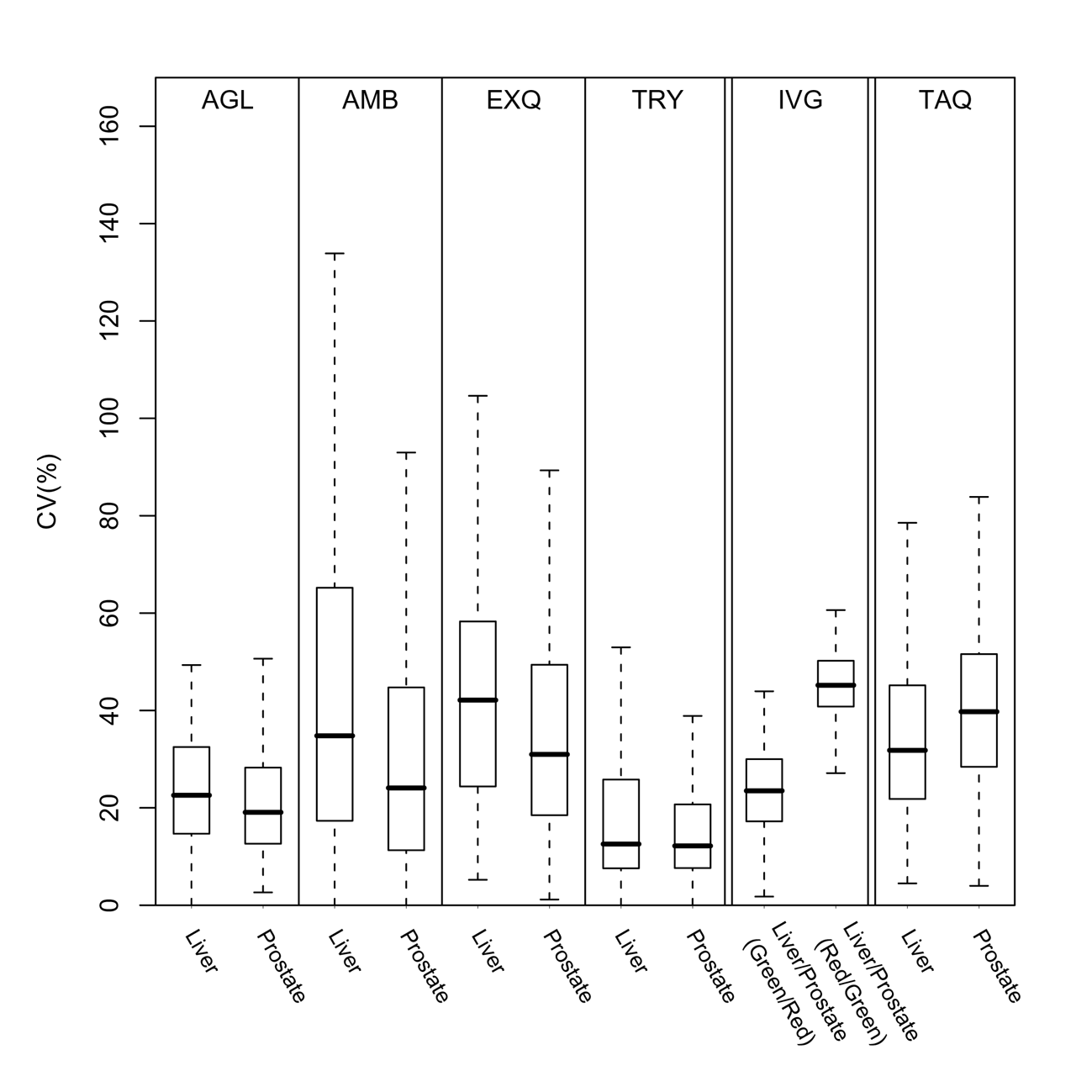

Supplement: Figure S6 — Effect of normalization on the coefficient of variation In contrast to Spearman's correlation coefficients in Figure S3, the coefficients of variation (CV) were drastically improved after the quantile normalization within the same replicates. The median values of CV in Figure S3 were significantly lower than those in Figure 3 (paired t-test, p = 0.03813). The CV of AGL and TRY were within the range of CV demonstrated in the original MAQC project paper. (5.07 MB TIF) [file pone.0005540.s006.tif]

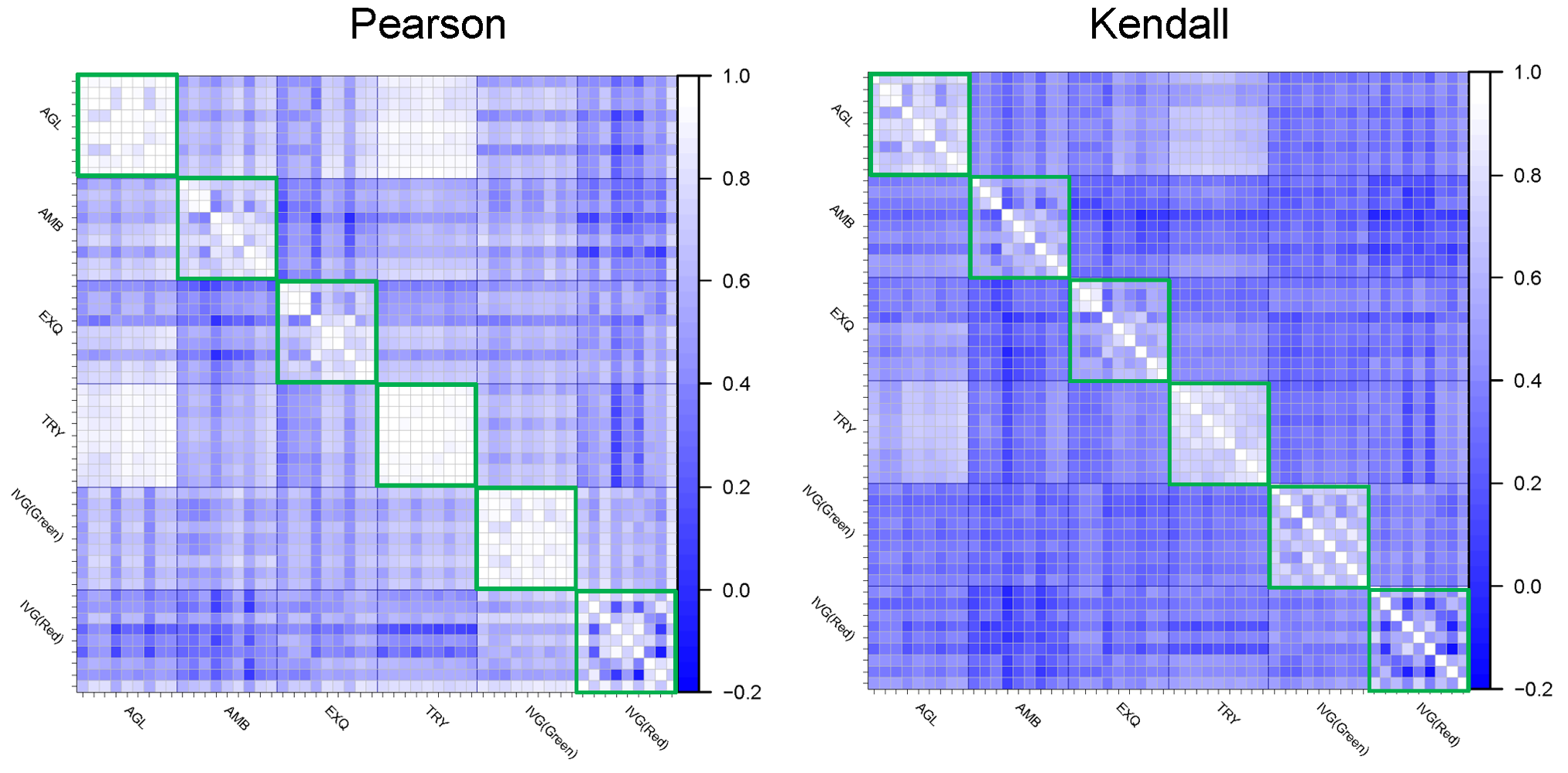

Supplement: Figure S7 — Correlation of log-ratios between intra- and inter-platform replications. Heatmaps of Pearson's correlation coefficients and Kendall's rank correlation coefficients. Both heatmaps had a similar pattern to heatmaps using Spearman's correlation coefficients in Figure 3. (6.08 MB TIF) [file pone.0005540.s007.tif]
