# Supplementary material for: Intra-Platform Repeatability and Inter-Platform Comparability of MicroRNA Microarray Technology
Source: PLoS One. 2009 May 14;4(5):e5540. doi: 10.1371/journal.pone.0005540 (PMC2677665; doi:10.1371/journal.pone.0005540)
Supplement: Table S1 — Skewness and Kurtosis of microRNA microarray data distribution Skewness and kurtosis of each data set was calculated using all expression data or non-zero log2 data of 309 microRNAs. A symmetric distribution has 0 skewness. A distribution with positive skew has a longer right tail, while a distribution with negative skew has a longer left tail. The kurtosis of the normal distribution is 3. A high kurtosis distribution has a sharper peak and longer, fatter tails, while a low kurtosis distribution has a more rounded peak and shorter thinner tails. This table demonstrated that microRNA microarray data tend to have a positive skewness. (0.03 MB DOC) [file pone.0005540.s008.doc]

Table S1

Skewness and Kurtosis of microRNA microarray data distribution

|  | | Tissue | AGL | AMB | EXQ | TRY | IVG (green) | IVG (Red) | TAQ |
| --- | --- | --- | --- | --- | --- | --- | --- | --- | --- |
| Skewness | detected | Liver | 0.401 | 0.250 | -0.012 | 0.700 | 1.072 | 0.755 | -0.086 |
|  |  | Prostate | 0.269 | -0.015 | 0.030 | 0.531 | 0.651 | 1.250 | -0.276 |
|  | all | Liver | 1.232 | 1.239 | 0.879 | 1.227 | 1.151 | 0.767 | 0.011 |
|  |  | Prostate | 0.685 | 0.973 | 0.045 | 0.614 | 0.676 | 1.372 | -0.356 |
| Kurtosis | detected | Liver | 1.957 | 1.798 | 2.001 | 2.204 | 3.013 | 2.418 | 1.939 |
|  |  | Prostate | 2.118 | 1.851 | 2.214 | 2.404 | 2.384 | 3.580 | 2.142 |
|  | all | Liver | 3.276 | 3.157 | 2.346 | 3.270 | 3.428 | 2.593 | 1.764 |
|  |  | Prostate | 2.247 | 2.475 | 1.928 | 2.427 | 2.577 | 4.207 | 2.151 |
