# Supplementary material for: Intra-Platform Repeatability and Inter-Platform Comparability of MicroRNA Microarray Technology
Source: PLoS One. 2009 May 14;4(5):e5540. doi: 10.1371/journal.pone.0005540 (PMC2677665; doi:10.1371/journal.pone.0005540)
Supplement: Table S2 — Rank correlation coefficients of log-ratios between intra- and inter-platforms of microRNA microarray. For rank correlation calculation, we used data of detected microRNAs that meet the detection criteria of each manufacturer. Both prostate and liver samples have triplicated data sets. Thus, 9 ( = 3×3) sets of log-ratios (prostate/liver) of microRNAs were generated. For intra-platform correlation, rank correlation coefficients of 36 ( = 9×8÷2) combinations were calculated, whereas, 81 ( = 9×9) coefficients were calculated for inter-platform correlation. Upper values: Spearman's correlation coefficients, Lower values: 95% confidence intervals. (0.03 MB DOC) [file pone.0005540.s009.doc]

|  | AGL | AMB | EXQ | TRY | IVG (Green) | IVG (Red) |
| --- | --- | --- | --- | --- | --- | --- |
| AGL | 0.862 0.828-0.897 | 0.539 0.515-0.564 | 0.594 0.570-0.618 | 0.785 0.765-0.805 | 0.448 0.433-0.463 | 0.470 0.444-0.496 |
| AMB | 0.539 0.515-0.564 | 0.676 0.618-0.733 | 0.343 0.315-0.371 | 0.461 0.432-0.49 | 0.376 0.357-0.396 | 0.259 0.23-0.287 |
| EXQ | 0.594 0.570-0.618 | 0.343 0.315-0.371 | 0.723 0.674-0.773 | 0.514 0.497-0.53 | 0.420 0.399-0.441 | 0.476 0.448-0.503 |
| TRY | 0.785 0.765-0.805 | 0.461 0.432-0.490 | 0.514 0.497-0.530 | 0.906 0.893-0.919 | 0.453 0.435-0.470 | 0.479 0.442-0.516 |
| IVG  (Green) | 0.448 0.433-0.463 | 0.376 0.357-0.396 | 0.420 0.399-0.441 | 0.453 0.435-0.470 | 0.831 0.798-0.864 | 0.540 0.518-0.561 |
| IVG  (Red) | 0.470 0.444-0.496 | 0.259 0.230-0.287 | 0.476 0.448-0.503 | 0.479 0.442-0.516 | 0.540 0.518-0.561 | 0.575 0.481-0.668 |
