# Supplementary material for: Intra-Platform Repeatability and Inter-Platform Comparability of MicroRNA Microarray Technology
Source: PLoS One. 2009 May 14;4(5):e5540. doi: 10.1371/journal.pone.0005540 (PMC2677665; doi:10.1371/journal.pone.0005540)
Supplement: Table S3 — List agreement of differentially expressed microRNA This table showed percentage of concordance in detecting differentially expressed microRNAs. The values in the upper portion of cells reflects the mean percent overlap of microRNAs on the list for the platform X (listed in column) that are also present of the list for the platform Y (listed in row), whereas the values in the lower portion were 95% confidence intervals of the mean percentage. A higher value indicates a high percent overlap between the microRNA lists at both platforms. A lower value indicates a low percent overlap, suggesting that most microRNAs identified in platform X were not identified in platform Y. Therefore, the table is asymmetric and not complementary. (0.03 MB DOC) [file pone.0005540.s010.doc]

|  | AGL | AMB | EXQ | TRY | IVG (Green) | IVG (Red) |
| --- | --- | --- | --- | --- | --- | --- |
| AGL | 0.910 0.890-0.930 | 0.634 0.617-0.652 | 0.609 0.593-0.625 | 0.695 0.680-0.709 | 0.380 0.362-0.398 | 0.386 0.369-0.403 |
| AMB | 0.501 0.480-0.521 | 0.690 0.656-0.725 | 0.392 0.375-0.409 | 0.448 0.428-0.467 | 0.338 0.326-0.350 | 0.249 0.234-0.264 |
| EXQ | 0.820 0.790-0.849 | 0.666 0.641-0.691 | 0.840 0.805-0.875 | 0.791 0.758-0.824 | 0.431 0.412-0.45 | 0.574 0.536-0.611 |
| TRY | 0.856 0.846-0.866 | 0.699 0.679-0.719 | 0.720 0.707-0.732 | 0.933 0.922-0.945 | 0.405 0.388-0.422 | 0.534 0.518-0.550 |
| IVG (Green) | 0.437 0.417-0.457 | 0.499 0.480-0.518 | 0.373 0.355-0.391 | 0.378 0.361-0.395 | 0.865 0.846-0.883 | 0.198 0.184-0.212 |
| IVG (Red) | 0.512 0.485-0.538 | 0.419 0.394-0.444 | 0.548 0.523-0.572 | 0.572 0.549-0.596 | 0.229 0.211-0.248 | 0.839 0.820-0.858 |
