# Supplementary material for: Intra-Platform Repeatability and Inter-Platform Comparability of MicroRNA Microarray Technology
Source: PLoS One. 2009 May 14;4(5):e5540. doi: 10.1371/journal.pone.0005540 (PMC2677665; doi:10.1371/journal.pone.0005540)
Supplement: Table S4 — Accession numbers of microarray data All microarray data were registered into NCBI's Gene Expression Omunibus (GEO) database (http://www.ncbi.nlm.nih.gov/projects/geo/). All data were available to public on March 30, 2009. (0.04 MB DOC) [file pone.0005540.s011.doc]

| Platforms | GSE | GPL | Accession | |
| --- | --- | --- | --- | --- |
|  |  |  | Liver | Prostate |
| Agilent | GSE13860 | GPL7718 | GSM347709 | GSM347712 |
|  |  |  | GSM347710 | GSM347713 |
|  |  |  | GSM347711 | GSM347714 |
| Ambion |  | GPL7719 | GSM347715 | GSM347718 |
|  |  |  | GSM347716 | GSM347719 |
|  |  |  | GSM347717 | GSM347720 |
| Exiqon |  | GPL7720 | GSM347721 | GSM347724 |
|  |  |  | GSM347722 | GSM347725 |
|  |  |  | GSM347723 | GSM347726 |
| Toray |  | GPL7766 | GSM349858 | GSM349861 |
|  |  |  | GSM349859 | GSM349862 |
|  |  |  | GSM349860 | GSM349863 |
|  |  |  | Liver/Prostate_Green/Red | Liver/Prostate_Red/Green |
| Invitrogen |  | GPL7721 | GSM347727 | GSM347730 |
|  |  |  | GSM347728 | GSM347731 |
|  |  |  | GSM347729 | GSM347732 |
